# GSK-3β regulates the endothelial-to-mesenchymal transition via reciprocal crosstalk between NSCLC cells and HUVECs in multicellular tumor spheroid models

**DOI:** 10.1186/s13046-019-1050-1

**Published:** 2019-02-01

**Authors:** Se-Hyuk Kim, Yeonhwa Song, Haeng Ran Seo

**Affiliations:** Cancer Biology Laboratory, Institut Pasteur Korea, 16, Daewangpangyo-ro 712 beon-gil, Bundang-gu, Seongnam-si, Gyeonggi-do 13488 Republic of Korea

**Keywords:** NSCLC (non-small-cell lung cancer) cells, HUVEC (human umbilical vein endothelial cells), Multicellular tumor spheroids (MCTS), EndMT (endothelial-to-mesenchymal transition), Chemoresistance, GSK-3β(glycogen synthase kinase -3β)

## Abstract

**Background:**

Chemotherapy used for patients with unresectable lung tumors remains largely palliative due to chemoresistance, which may be due to tumor heterogeneity. Recently, multiple studies on the crosstalk between lung cancer cells and their tumor microenvironment (TME) have been conducted to understand and overcome chemoresistance in lung cancer.

**Methods:**

In this study, we investigated the effect of reciprocal crosstalk between lung cancer cells and vascular endothelial cells using multicellular tumor spheroids (MCTSs) containing lung cancer cells and HUVECs.

**Results:**

Secretomes from lung cancer spheroids significantly triggered the endothelial-to-mesenchymal transition (EndMT) process in HUVECs, compared to secretomes from monolayer-cultured lung cancer cells. Interestingly, expression of GSK-3β-targeted genes was altered in MCTSs and inhibition of this activity by a GSK-3β inhibitor induced reversion of EndMT in lung tumor microenvironments. Furthermore, we observed that HUVECs in MCTSs significantly increased the compactness of the spheroids and exhibited strong resistance against Gefitinib and Cisplatin, relative to fibroblasts, by facilitating the EndMT process in HUVECs. Subsequently, EndMT reversion contributed to control of chemoresistance, regardless of the levels of soluble transforming growth factor (TGF)-β. Using the MCTS xenograft mouse model, we demonstrated that inhibition of GSK-3β reduces lung cancer volume, and in combination with Gefitinib, has a synergistic effect on lung cancer therapy.

**Conclusion:**

In summary, these findings suggest that targeting EndMT through GSK-3β inhibition in HUVECs might represent a promising therapeutic strategy for lung cancer therapy.

**Electronic supplementary material:**

The online version of this article (10.1186/s13046-019-1050-1) contains supplementary material, which is available to authorized users.

## Introduction

Lung cancer ranks highest in terms of both incidence and mortality in the world. Despite advances in our knowledge of molecular mechanisms and the introduction of multiple new therapeutic lung cancer agents, the dismal 5-year survival rate (11–15%) remains relatively unaltered [1–3]. Lung cancers are comprised of two major histological types: small-cell lung cancer (SCLC) and non-small-cell lung cancer (NSCLC; i.e., adenocarcinoma, squamous cell carcinoma, and large cell carcinoma). NSCLC comprises 85% of lung cancer cases, and about 40% are unresectable [4].

The clinical success of oncogene-targeted therapy in specific subsets of patients with lung cancer, such as those with activating mutations in the epidermal growth factor receptor (EGFR), has heralded a new era of precision medicine for cancer that holds great promise for improving patient survival and quality of life [5–10]. However, tumor progression often occurs via the emergence of the EGFR T790 M resistance mutation during the treatment of EGFR-mutant lung adenocarcinomas patients with first-generation EGFR tyrosine kinase inhibitors (TKIs; Erlotinib, Gefitinib) [10, 11]. This observation prompted the development of second- and third-generation irreversible EGFR inhibitors (Afatinib and Osimertinib, respectively) with activity against EGFR T790 M [10, 12, 13]. Chemotherapy used for patients with unresectable lung tumors remains largely palliative, due to chemoresistance, which is possibly due to tumor heterogeneity [14]. Hence, a deeper knowledge of the crosstalk between tumor cells and their tumor microenvironment (TME) is needed to fully understand the development, progression, and chemoresistance of lung cancer.

The TME represents a milieu that enables tumor cells to acquire the hallmarks of cancer. The TME is heterogeneous in composition and consists of cellular components, growth factors, proteases, and the extracellular matrix [15, 16]. Concerted interactions between genetically altered tumor cells and genetically stable intratumoral stromal cells result in an “activated/reprogrammed” stroma that promotes carcinogenesis by contributing to inflammation, immune suppression, therapeutic resistance, and generates premetastatic niches that support the initiation and establishment of distant metastasis.

The lungs present a unique milieu in which tumors progress in collusion with the TME, as evidenced by regions of aberrant angiogenesis, desmoplasia, acidosis and hypoxia [17]. The TME also contributes to immune suppression, induces epithelial-to-mesenchymal transition (EMT) and endothelial-to-mesenchymal transition (EndMT), and diminishes the efficacy of chemotherapies [18]. Thus, the TME has begun to emerge as the “Achilles heel” of the disease, and constitutes an attractive target for anticancer therapy [19]. Drugs targeting the components of the TME are making their way into clinical trials. The accumulation of activated fibroblasts, which are termed peritumoral fibroblasts or cancer-associated fibroblasts (CAFs), within lung cancer is widely accepted [20]. CAFs are derived from pericytes and smooth muscle cells from the vasculature, from bone marrow-derived mesenchymal cells, or during EMT or EndMT [21–23].

In particular, the EndMT is characterized by the loss of endothelial marker expression and the acquisition of mesenchymal or fibroblastic phenotypes consisting of the production of smooth muscle actin (SMA), fibroblast-specific protein 1 (FSP1), and type I collagen (COL I), resulting in cells that have invasive and migratory potential [24] . Recently, it has been proposed that modulation of the EndMT may provide an effective therapeutic strategy for various fibrotic diseases [25, 26]. The EndMT has also been reported to play a critical role in pulmonary hypertension, with accumulation of mesenchymal-like cells in obstructive pulmonary vascular lesions [27].

To date, monolayer culture-based assay models have dominated cancer biology and preclinical cancer drug discovery efforts. However, these models fail to predict in vivo efficacy, contributing to a lower success rate in translating a new investigational drug to clinical approval. Recently, scientists have highlighted the need for complex three-dimensional (3D) cell culture systems in oncology research, because tumor spheroids strikingly mirror the 3D in vivo context. These 3D cell culture systems also model therapeutically relevant pathophysiological gradients of in vivo tumors, such as pH, oxygen, nutrient, and drug concentrations [28]. Hence, multicellular 3D culture and interaction with stromal components are considered essential elements in establishing a “more clinically relevant” tumor model [29–31].

In this study, we sought to identify EndMT mechanisms induced by crosstalk between lung cancer and vascular endothelial cells in lung cancer progression and chemoresistance, using diverse forms of multicellular tumor spheroids (MCTSs). Based on our observations, we also discuss future directions in therapeutic opportunities.

## Materials and methods

### Cell line and cell culture

NCI-H460, A549, and SK-MES-1 cells were obtained from the Korean Cell Line Bank. Human umbilical vein endothelial cells (HUVECs) were obtained from Lonza (Basel, Switzerland) and WI38 cells (human fibroblasts) were purchased from the American Type Culture Collection (AATC; Manassas, VA, USA). The cells were maintained at 37 °C in a humidified atmosphere of 5% CO_2_. H460 cells were cultured in Roswell Park Memorial Institute medium (RPMI 1640; Welgene, Korea) supplemented with 10% fetal bovine serum (FBS; Gibco, Grand Island, NY, USA), 1× penicillin-streptomycin (P/S; Gibco, Grand Island, NY, USA) (complete medium). A549 cells were cultured in Dulbecco’s Modified Eagle medium (DMEM; Welgene, Korea) supplemented with 10% FBS and 1× P/S. For HUVECs, endothelial basal medium (EBM) was purchased from Lonza.

### Conditioned medium experiment and immunofluorescence staining

NCI-H460 cells were cultured in 2D or 3D conditioned medium using the same number of cells and amount of medium for 3 days. Conditioned medium(CM) was collected when the cells reached 70–90% confluence in 2D culture, and passed through a 0.45-μM pore filter (Millipore, Billerica, MA, USA) to eliminate debris. CM from both 2D and 3D cultures was mixed with the original medium at various ratios. HUVECs were seeded at a density of 2 × 10^3^ cells/well in a 384-well plate (Greiner Bio-one, Monroe, NC, USA) and treated with 40 μl of 2D- or 3D-CM for 24 and 48 h. The HUVECs were fixed in 4% paraformaldehyde (PFA; Biosesang, Korea) for 10 min at room temperature (RT), washed with Dulbecco’s Phosphate-Buffered Saline (DPBS) twice, and then permeabilized with 0.1% Triton X-100 (Sigma-Aldrich, St. Louis, MO, USA) in PBS for 10 min at room temperature. The following primary antibodies were used: mouse monoclonal anti-cluster of differentiation 31 (CD31; Cell Signaling Technology, Danvers, MA, USA) and rabbit polyclonal anti-alpha-smooth muscle actin (α-SMA; Abcam, Cambridge, UK). The primary antibodies were incubated for 16 h at 4 °C, and then washed three times for 5 min with PBS. The secondary antibodies used for staining were: goat anti-mouse Alexa® Fluor 488 and goat anti-rabbit Alexa® Fluor 546 (Invitrogen Life Technologies, Grand Island, NY, USA). Secondary antibodies were incubated for 1 h at room temperature and washed three times for 5 min with PBS. Fluorescent images were captured according to the optimal excitation and emission wavelengths of each probe. To capture entire images, we collected 25 image fields, starting at the center of the well, from each well using the high content screening (HCS) system with a 10× objective.

### Generation of tumor spheroid

To generate spheroids, cells suspended in complete medium were seeded at a density of 6 × 10^3^ cells/well in 96-well round-bottomed ultra-low attachment microplates (Corning B.V. Life Sciences, Amsterdam, Netherlands). The plates were incubated for 3 days at 37 °C in a humidified atmosphere of 5% CO_2_. To generate MCTSs containing various cell types, lung cancer cells, and stromal cells (WI38 and HUVECs) were mixed at a 5:5 ratio.

### Cell death detection in spheroid

For drug treatment, lung cancer cells (NCI-H460, A549, and SK-MES-1) and stromal cells (WI38 and HUVEC) were seeded at a density of 6 × 10^3^ cells/well in 96-well round-bottomed ultra-low attachment microplates. After 1 day, 10 or 20 μM of Gefitinib and Cisplatin (all from Sigma-Aldrich, St. Louis, MO, USA) were added to the spheroids for 2 days, and then spheroid cell death was detected using the cell-impermeant viability indicator ethidium homodimer-1 (EthD-1; Invitrogen Life Technologies, Grand Island, NY, USA). EthD-1 is a high-affinity nucleic acid stain that fluoresces weakly until bound to DNA, whereupon it emits red fluorescence (excitation/emission maxima ~ 528/617 nm). Spheroids were incubated in 4 μM EthD-1 in complete medium for 30 min in a 37 °C incubator, and images were obtained and the intensity of EthD-1 fluorescence measured using the Operetta® High Content Screening System (Perkin Elmer).

### Drug treatment and western blot

Lung cancer cells (NCI-H460 and A549) were cultured with HUVECs at a density of 1 × 10^6^ cells/well in an ultra-low attachment 6-well plate (Corning B.V. Life Sciences, Amsterdam, Netherlands), and were simultaneously treated with various drugs, including CHIR-99021 (Selleckchem, Houston, TX, USA), SB-216763, MASB, and IWR-1 (all from Sigma-Aldrich, St. Louis, MO, USA). After 1 day, 3D-cultured cells were harvested and then subjected to western blotting. Cells were lysed using radioimmunoprecipitation assay (RIPA) buffer (3 M, Seoul, Korea) and boiled with 5× sample buffer for 10 min. Cell lysates were separated by 8–15% sodium dodecyl sulfate-polyacrylamide gel electrophoresis (SDS-PAGE) and transferred to a nitrocellulose (NC) membrane. A blocking step was performed for 1 h at room temperature with 5% non-fat dry milk in Tris-buffered saline/Tween 20 (TBST) buffer. Blotting with antibodies was performed overnight at 4 °C. The primary antibodies were: mouse monoclonal anti-CD31 (89C2), rabbit monoclonal anti-VE-Cadherin (D87F2), mouse monoclonal anti-vimentin (RV202), rabbit polyclonal anti-phospho-GSK-3β (Ser9), rabbit monoclonal anti-GSK-3β (27Χ10), rabbit monoclonal anti-cleaved PARP (D64E10) (all from Cell Signaling Technology, Danvers, MA, USA), rabbit monoclonal anti-α-SMA (E184), rabbit polyclonal anti-TGF-β1, rabbit polyclonal anti-phospho-GSK-3β (Tyr216), rabbit polyclonal anti-CD31 (all from Abcam, Cambridge, UK), and mouse monoclonal anti-β-Actin (Sigma-Aldrich, St. Louis, MO, USA).

### Enzyme-linked immunosorbent assay (ELISA)

Lung cancer cells (NCI-H460 and A549) were seeded at a density of 1 × 10^6^ cells/well in a 100-mm dish or ultra-low attachment 6-well plate. After 2 days, 2D- or 3D-CM was collected and filtered with a 0.45-μM pore filter to eliminate debris. Relative transforming growth factor beta 1 (TGF-β1) secretion was quantified from conditioned medium of lung cancer cells using an ELISA kit according to the manufacturer’s instructions (R&D Systems, Minneapolis, MN, USA).

### Akt pathway phosphorylation array

NCI-H460 cells were cultured with HUVECs seeded at a density of 1 × 10^6^ cells/well in a 100-mm dish or ultra-low attachment 6-well plate. After 1 day, 2D or 3D-cultured cells were harvested and lysed using the cell lysis buffer provided in the human/mouse Akt Pathway Phosphorylation Array C1 (RayBiotech, Norcross, GA, USA). Protein extraction was performed according to the manufacturer’s recommended protocol.

### Microarray analysis

Global gene expression analysis was performed using Affymetrix GeneChip® Human Gene 2.0 ST Arrays. Total RNA from NCI-H460 cells co-cultured with HUVECs in 2D or 3D culture conditions was isolated using the RNeasy Mini kit (Qiagen, Hilden, Germany). RNA quality was assessed using an Agilent 2100 Bioanalyser using the RNA 6000 Nano Chip (Agilent Technologies), and the quantity was determined using a Nanodrop-1000 Spectrophotometer (Thermo Scientific). We used 300 μg of each RNA sample as input for the Affymetrix procedure, as recommended in the manufacturer’s protocol (http://www.affymetrix.com). Briefly, 300 ng of total RNA from each sample was converted to double-stranded cDNA using a random hexamer incorporating a T7 promoter, and amplified RNA (cRNA) was generated from the double-stranded cDNA template though an in vitro transcription (IVT) reaction and purified using the Affymetrix sample cleanup module. cDNA was regenerated through randomly primed reverse transcription using a dNTP mix containing dUTP. The cDNA was then fragmented by uracil-DNA glycosylase (UDG) and apurinic/apyrimidinic endonuclease (APE 1) restriction enzymes, and end-labeled via a terminal transferase reaction incorporating a biotinylated dideoxynucleotide. Fragmented end-labeled cDNA was hybridized to the GeneChip® Human Gene 2.0 ST array for 17 h at 45 °C and 60 rpm, as described in the Gene Chip Whole Transcript (WT) Sense Target Labeling Assay Manual (Affymetrix). After hybridization, the chips were stained and washed in a Genechip Fluidics Station 450 (Affymetrix) and scanned using a Genechip Array scanner 3000 7G (Affymetrix). The expression intensity data were extracted from the scanned images using Affymetrix Command Console software, version 1.1, and stored as CEL files.

### Data analysis

The intensity values of CEL files were normalized to remove bias between the arrays (M1), using the Robust Multi-array Average (RMA) algorithm implemented in the Affymetrix Expression Console software (version 1.3.1.) (http://www.affymetrix.com). The normalized data were imported into the programming environment R (version 3.0.2) and overall signal distributions of each array were compared by plotting using tools available from the Bioconductor Project (http://www.bioconductor.org) (M2) to confirm normalization. After confirming whether the data were properly normalized, differentially expressed genes (DEGs) that showed differences of over 1.5-fold between the average signal values of the control groups and treatment groups were manually selected. Finally, using the web-based tool DAVID (the Database for Annotation, Visualization, and Integrated Discovery), DEGs were functionally annotated and classified based on gene function information to reveal the regulatory networks that involve these genes (http://david.abcc.ncifcrf.gov) (M4).

### Immunohistochemistry

Tumor tissues were fixed in 4% PFA, cut into 4-μm paraffin-embedded sections, and stained with hematoxylin and eosin (H&E) and Masson’s trichrome. For immunohistochemistry, the tumor tissues were fixed overnight in 4% PFA, embedded in paraffin, and sectioned. After deparaffinization and dehydration, antigen retrieval was performed by boiling the sections in 10 mM citric acid buffer (pH 6.0) for 15 min. Antibodies against anti-CD31 (ab28364; Abcam) and Ki67 (ab15580; Abcam) were used in the immunohistochemistry studies. The secondary antibody was anti-IgG conjugated with Alexa® Fluor 546 (Invitrogen), and the stained cells were viewed under a fluorescence microscope (Nikon, Tokyo, Japan).

### Xenograft mouse model

Lung cancer cells (NCI-H460) were cultured with HUVECs seeded at a density of 1 × 10^6^ cells/well in an ultra-low attachment 6-well plate (Corning B.V. Life Sciences, Amsterdam, Netherlands). After 2 days, MCTSs were implanted subcutaneously in male BALB/c-nu mice and allowed to grow to a tumor volume of 100–200 mm. Tumor-bearing mice were randomized into the following four groups of six mice each; Group 1, control with normal saline (N/S); Group 2, treated with CHIR-99021 (Selleckchem, Houston, TX, USA), 16 mg/kg, intraperitoneal injection (i.p.); Group 3, treated with Gefitinib (Selleckchem, Houston, TX, USA), 50 mg/kg, oral injection (p.o.); Group 4, treated with CHIR-99021 and Gefitinib, respectively. All drugs were administered 3 days a week for 2 weeks. All treated mice were sacrificed on day 28, and tumors were resected for further histological evaluation.

### Statistical analysis

All experiments were performed at least three times. The data are shown as the mean ± SD. Statistical analysis was conducted using Microsoft Excel. Categorical data were compared using two-tailed Student’s t-test. *P*-values < 0.05 were considered statistically significant.

## Results

### Secretomes from NSCLC spheroids induced EndMT

To ascertain our hypothesis, we sought to confirm whether secretomes from different culture systems of lung cancer cells affected on EndMT of HUVEC cells have collected conditioned medium(CM) from monolayer (2D) cultured H460 cells or 3D cultured H460 cells.

**Fig. 1:**
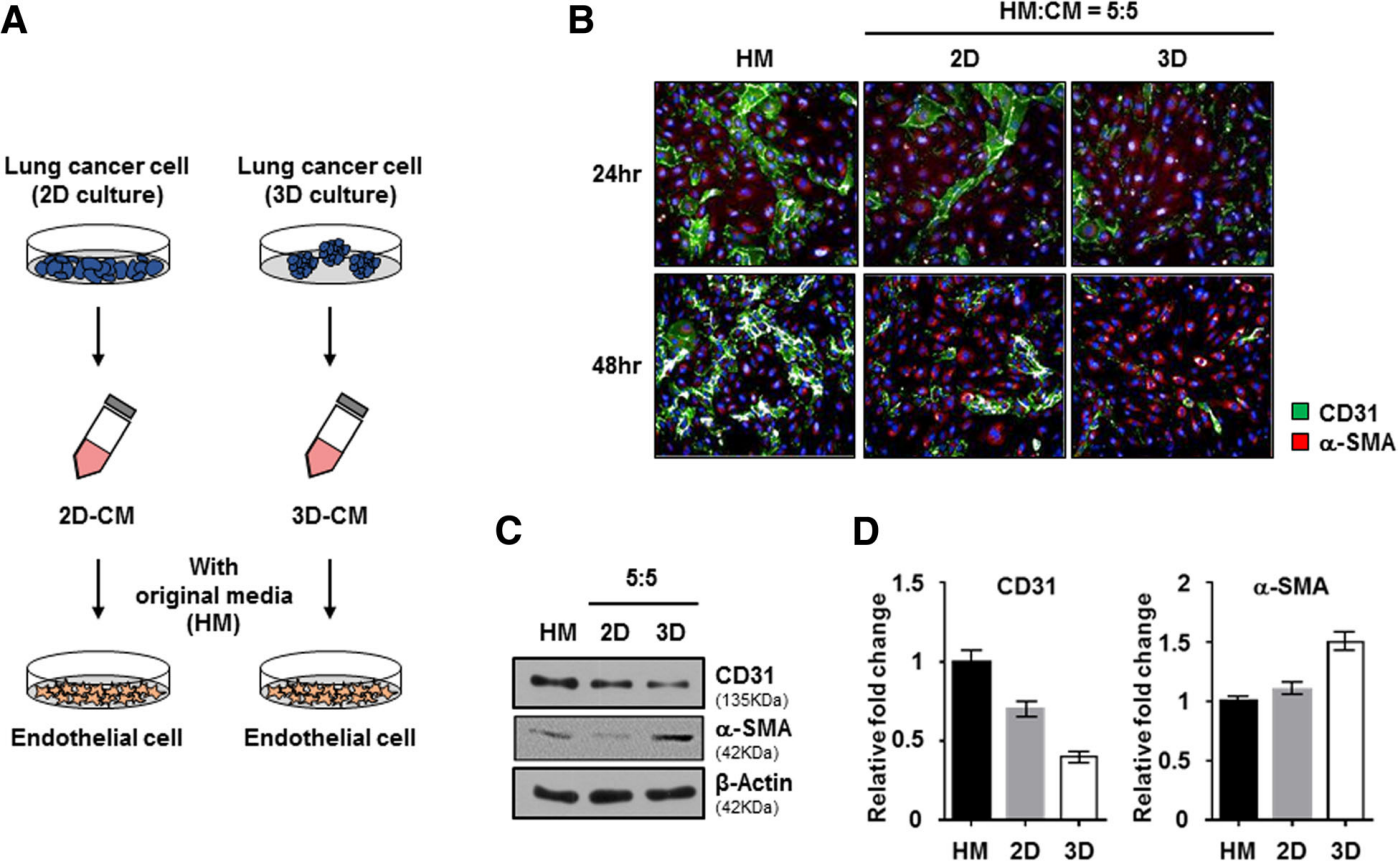
Secretomes from NSCLC spheroids induced endothelial-to-mesenchymal transition. **a** Experimental schematic of secretomes from NSCLC. NCI-H460 cells were cultured under 2D and 3D conditions using the same number of cells with the same amount of media. After 3 days, the conditioned medium (CM) was mixed with HUVEC original media at various ratios, and then used to treat HUVECs for 24 and 48 h. **b** Representative images of immunofluorescence staining for CD31 (green) and α-SMA (red) expression and nuclei (blue) in HUVECs treated with CM from 2D and 3D NCI-H460 cells at a ratio of 5:5. **c** HUVECs that were treated with CM from 2D and 3D NCI-H460 cells were harvested after 48 h incubation. HUVEC lysates were analyzed by western blotting using anti-CD31, anti-α-SMA, and anti-β-actin antibodies. **d** Quantification of relative expression levels of CD31 and α-SMA. Values were normalized to β-actin. Data are shown as mean ± SD from two independent experiments

2D cultured HUVEC cells were incubated with CMs from monolayer cultured H460 cells or 3D cultured H460 cells for 24 or 48 h (Fig. 1-a).

The transformation of HUVEC cells to a fibroblast-like morphology was more significant in the CM from the 3D cultured NSCLC than in the CM from the 2D cultured NSCLC. We selected two distinctive markers, CD31 as endothelial-specific marker and α-SMA as fibroblast marker used for detection of EndMT phenomena in HUVEC cells. Immunofluorescent staining analysis revealed that CM from 3D cultured H460 cells more sufficiently down regulated CD31 and upregulated α-SMA rather than CM from 2D cultured H460 cells in a dose- and time-dependent dependent manner (Fig. 1-b, Additional file 1: Figure S1). We also observed that the expression of CD31 in HUVEC cells was also relatively lower in CM from 3D cultured H460 cells than in CM from 2D cultured H460 cells (Fig. 1-c). At 24 h after incubation in CM from 3D cultured H460 cells, the level of α-SMA expression in HUVEC cells was significantly increased about 1.5-fold, whereas the level of CD31 expression was significantly decreased approximately 0.7-fold. On the other hand, we didn’t observed significant change of CD31 and α-SMA expression in HUVEC after incubation in CM from 2D cultured H460 cells (Fig. 1-d). These results suggested that 3D tumor microenvironments of NSCLC could induce phenotypic conversion from endothelial cells to form myofibroblast-like cells via the EndMT mechanism to form fibroblast-like cells.

### The interaction between HUVEC cells and NSCLC cells in MCTS facilitates the compactness of tumor spheroids through EndMT process

**Fig. 2:**
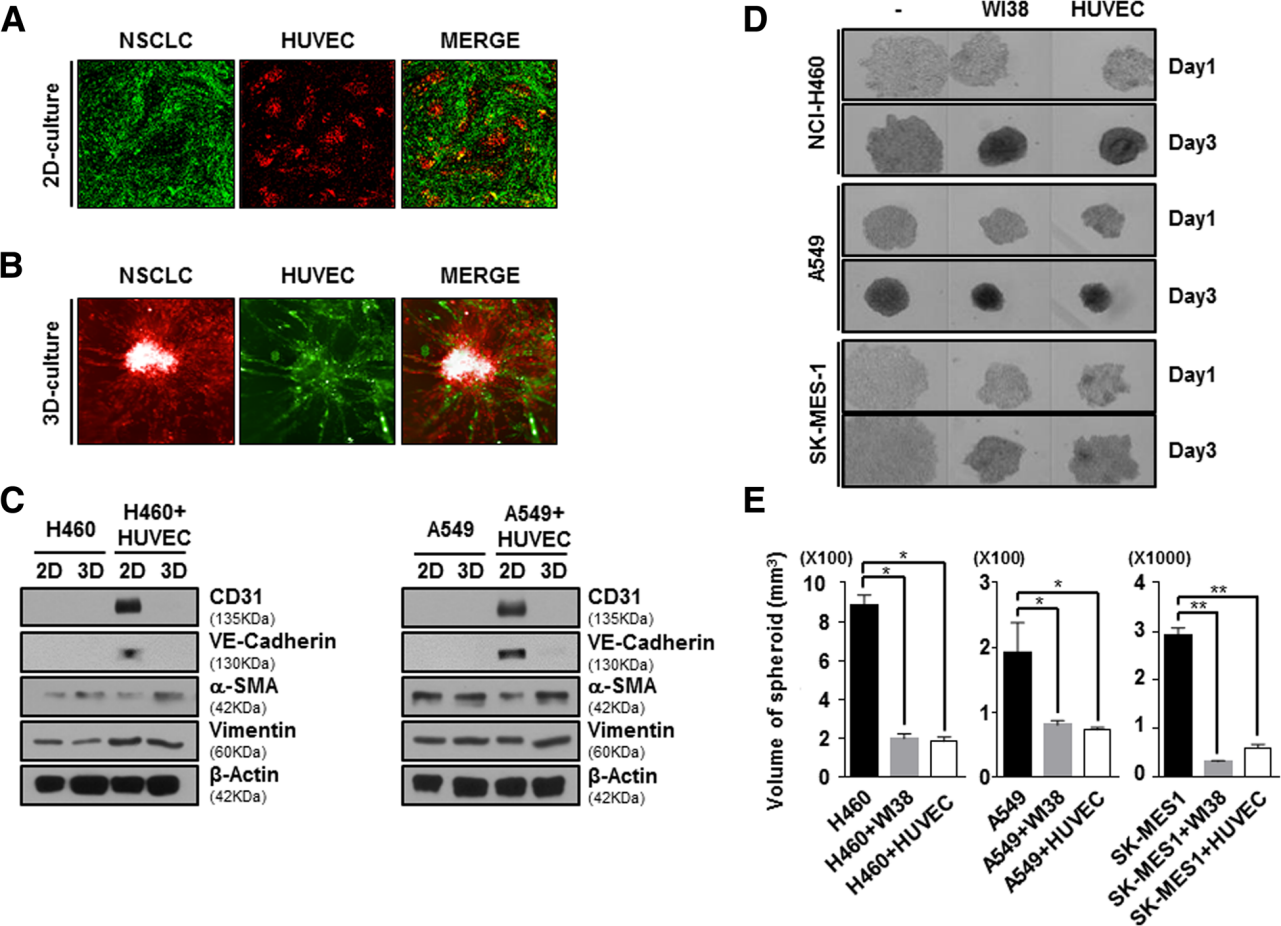
Interaction between NSCLC cells and HUVECs in MCTSs affects the compactness of tumor spheroids. **a** and **b** NSCLC, which is stained by DiO, and HUVECs stained by DiD were cultured for 3 days in 2D or 3D culture systems and then the images were obtained. **c** NCI-H460 or A549 cells were co-cultured with HUVECs in 2D and 3D culture systems at a ratio of 5:5 for 48 h. Lysates were analyzed by immunoblotting using indicated antibodies. **d** NCI-H460, A549, and SK-MES-1 cells were co-cultured with stromal cells (WI38 cells and HUVECs) at a ratio of 5:5 for 1 day and 3 days. All bright-field images of spheroids were obtained using the Operetta® High Content Screening System with a 10× objective. **e** To calculate the volume of spheroids, their long and short diameters were measured. Data are shown as mean ± SD from two independent experiments in triplicate. *P < 0.05, **P < 0.01 versus Control (NCI-H460, A549, or SK-MES-1)

To model tumor complexity and heterogeneity in vitro, we co-cultured with NSCLC and HUVEC cells in 2D or 3D culture systems instead of using conditioned media. To investigate whether co-culture with NSCLC and HUVEC cells in 2D or 3D systems displayed different crosstalk, we compared the distribution between NSCLC and HUVEC cells was observed. In 2D co-culture condition, NSCLC and HUVEC cells were scattered in their own way without co-localization (Fig. 2-a). On the contrary, 3D co-culture with NSCLC and HUVEC cells displayed plenty of co-localization. Furthermore, 3D co-culture with NSCLC and HUVEC cells showed sprouting of HUVEC cells (Fig. 2-b).

In order to certainly difference of EndMT activity between 2D and 3D co-culture systems, we also investigated the expression of CD31, VE-Cadherin and α-SMA against lysates form both co-culture with NSCLC (NCI-H460 or A549) and HUVEC cells. Unlike 2D co-culture with NSCLC and HUVEC cells, expression of CD31 and VE-Cadherin as endothelial cell marker was rarely detected in 3D co-culture with NSCLC and HUVEC cells. On the other hand, expression of α-SMA as mesenchymal cell marker was significantly increased in 3D co-culture with NSCLC and HUVEC cells rather than 2D co-culture with NSCLC and HUVEC cells, but not Vimentin (Fig. 2-c). These results indicated that multicellular tumor spheroids (MCTS) with NSCLC and HUVEC cells could induce transition of HUVEC cells from endothelial cells to myofibroblast-like cells.

To confirm whether HUVEC cells in MCTS have fibroblast-like properties, we formed MCTS with NSCLC cell lines, which are NCI-H460, A549 and SK-MES-1, were co-cultured with WI38 cells (human fibroblasts), and HUVEC cells. NCI-H460, A549 and SK-MES-1 cells loosely bound to each other and failed to show strong cell cohesion during the progress of spheroid assembly. However, we clearly observed a profound enhancement of spheroid compactness in spheroids with stromal cells and NSCLC cells relative to NSCLC-only spheroids. Intriguingly, co-culture with NSCLC and HUVEC cells in MCTS exhibited significantly induced enhancement of spheroid compactness and rigidness such as NSCLC-MCTS with WI38 cells (Fig. 2-d, e).

### Reciprocal crosstalk between NSCLC cells and HUVEC cells induces activation of GSK-3β in multicellular tumor spheroids models

**Fig. 3:**
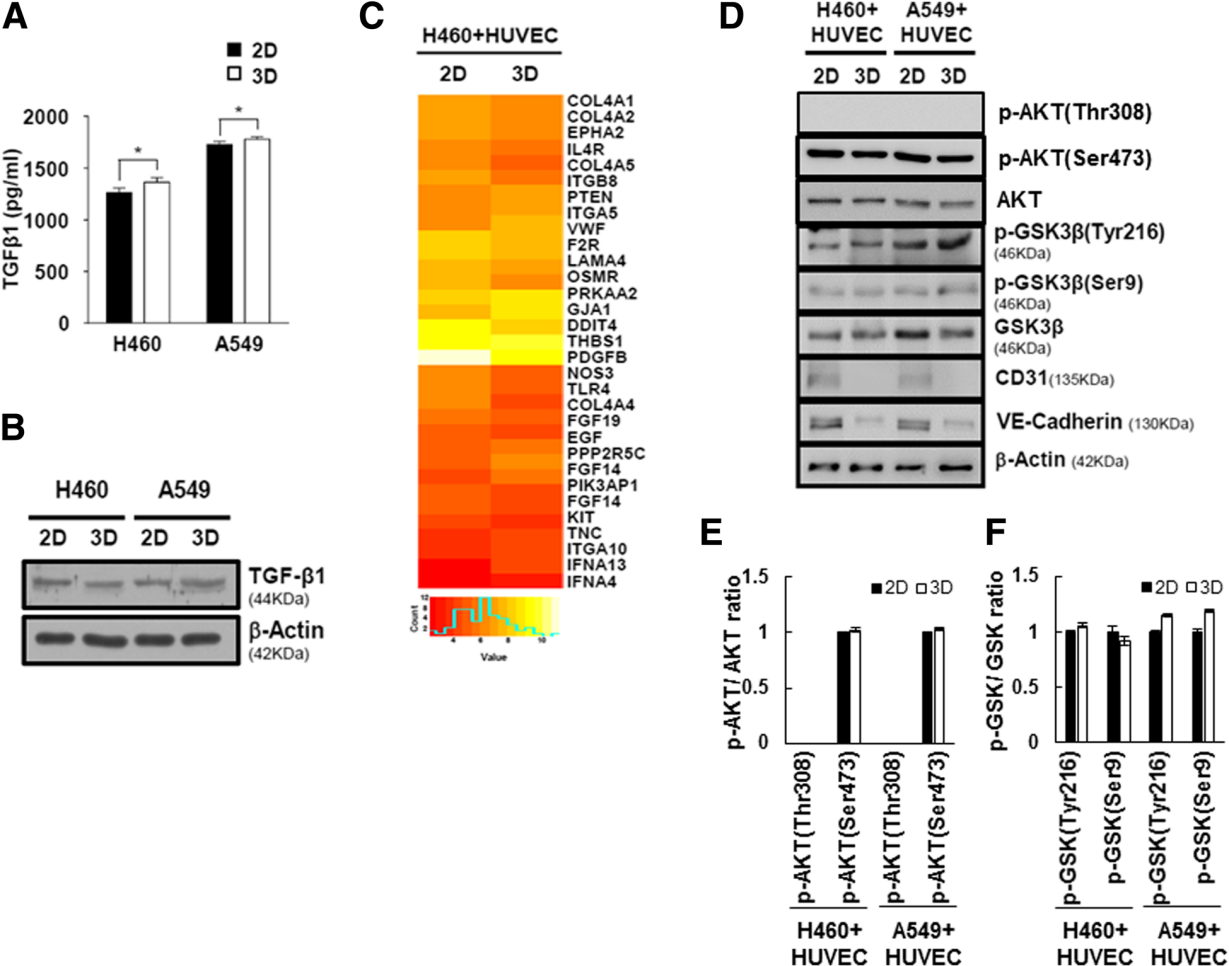
Reciprocal crosstalk between NSCLC cells and HUVECs induces activation of GSK-3β in multicellular tumor spheroid models. **a** NCI-H460 or A549 cells were cultured under 2D and 3D conditions for 2 days. The levels of TGF-β1 in culture supernatants were measured using ELISA. Results are presented as mean ± standard error of the means. *P < 0.05 versus 2D condition. **b** NCI-H460 or A549 cells were cultured in 2D and 3D culture systems for 2 days. Lysates were analyzed by immunoblotting using anti-TGF-β1 and anti-β-actin antibodies. **c** Gene expression heat map representing fold changes greater that 1.5 in samples from co-cultures of NCI-H460 cells with HUVECs under 2D and 3D conditions for 1 day. Categorization of Akt pathway-related genes. **d** The expression levels of GSK-3β and phosphorylated GSK-3β were determined by immunoblotting of samples from co-cultures of NCI-H460 cells or A549 cells with HUVECs under 2D and 3D conditions. **e** and **f** The ratio of p-AKT/AKT (**e)** and p-GSK-3β/ GSK-3β (**f**) was calculated

We wondered what kind of growth factors in CM from 3D cultured lung cancer cells involved process of EndMT in HUVEC cells. So, we additionally screened expression of growth factors in lysates from monolayer cultured NCI-H460 cells and NCI-H460 spheroids using human growth factor antibody array (Additional file 1: Figure S2A). However, there are no significant differences of expression pattern of growth factors between NCI-H460 cells and NCI-H460 spheroids (Fig. 3-a, b, Additional file 1: Figure S2B). Additionally, NCI-H460 spheroids and NCI-H460-MCTS grown together with NCI-H460 and HUVEC cells also did not make significant differences of level of growth factors (Additional file 1: Figure S2C). These results represented that increased EndMT activity is not connected with the levels of TGF-β in NSCLC spheroids.

To validate the change of gene expression profiling on 2D and 3D co-culture with NCI-H460 and HUVEC cells, we performed microarray analysis. A total of 3996 genes were differentially expressed between 2D and 3D samples, using a fold change of at least 1.5. Among these, 2180 genes showed increased expression in 3D samples, while 1816 genes showed reduced expression in 3D samples. A number of autocrine or paracrine signaling molecules can induce EndMT. Interestingly, AKT pathway-related genes were mostly downregulated, but the PTEN as tumor suppressor gene by negatively regulating AKT signaling pathway was increased in 3D co-culture with NCI-H460 and HUVEC cells (Fig. 3-c). The raw data from microarray was listed in the Additional File 2. To identify activities of AKT pathway proteins between co-cultured with NCI-H460 and HUVEC cells in 2D and 3D culture systems, we determined phosphorylation level of AKT and GSK-3β (Fig. 3-d). Although mRNA level of AKT pathway-related genes were changed in 3D co-culture with NCI-H460 and HUVEC cells, there was no significant difference in activation of AKT pathway between 2D and 3D samples.

GSK-3β is expressed ubiquitously and regulates diverse cellular process via over 40 substrates, such as proliferation, migration, and invasion-related proteins. Full activity of GSK-3β generally requires phosphorylation of Tyrosine (Try216), and conversely, phosphorylation of Serine (Ser9) inhibits GSK-3β activity. Interestingly, we found that status of phosphorylated GSK-3β at Tyr 216 were significantly increased in the 3D co-culture with NSCLC and HUVEC cells relative to 2D co-culture with NSCLC and HUVEC cells, but not phosphorylated GSK-3β at Ser 9 (Fig. 3-e and f). Therefore, we suggested that GSK-3β is activated through AKT independent signaling pathway in 3D co-culture with NSCLC and HUVEC cells.

### CHIR-99021, inhibitor of GSK-3β, suppresses EndMT process in NSCLC-MCTS with HUVEC

One of the most well-known substrates of GSK-3β is β-catenin, and GSK-3β is an important regulator of the Wnt/ β-catenin signaling pathway. We searched for small-molecule compounds that act against Wnt/β-catenin signaling to understand signal pathway of EndMT in 3D co-cultured system. MCTSs grown with NCI-H460 and HUVEC cells were incubated with CHIR-99021 and SB-216763 as inhibitors of GSK-3β, and also MSAB and IWR-1, inhibitors of β-catenin, treated for 24 h.

**Fig. 4:**
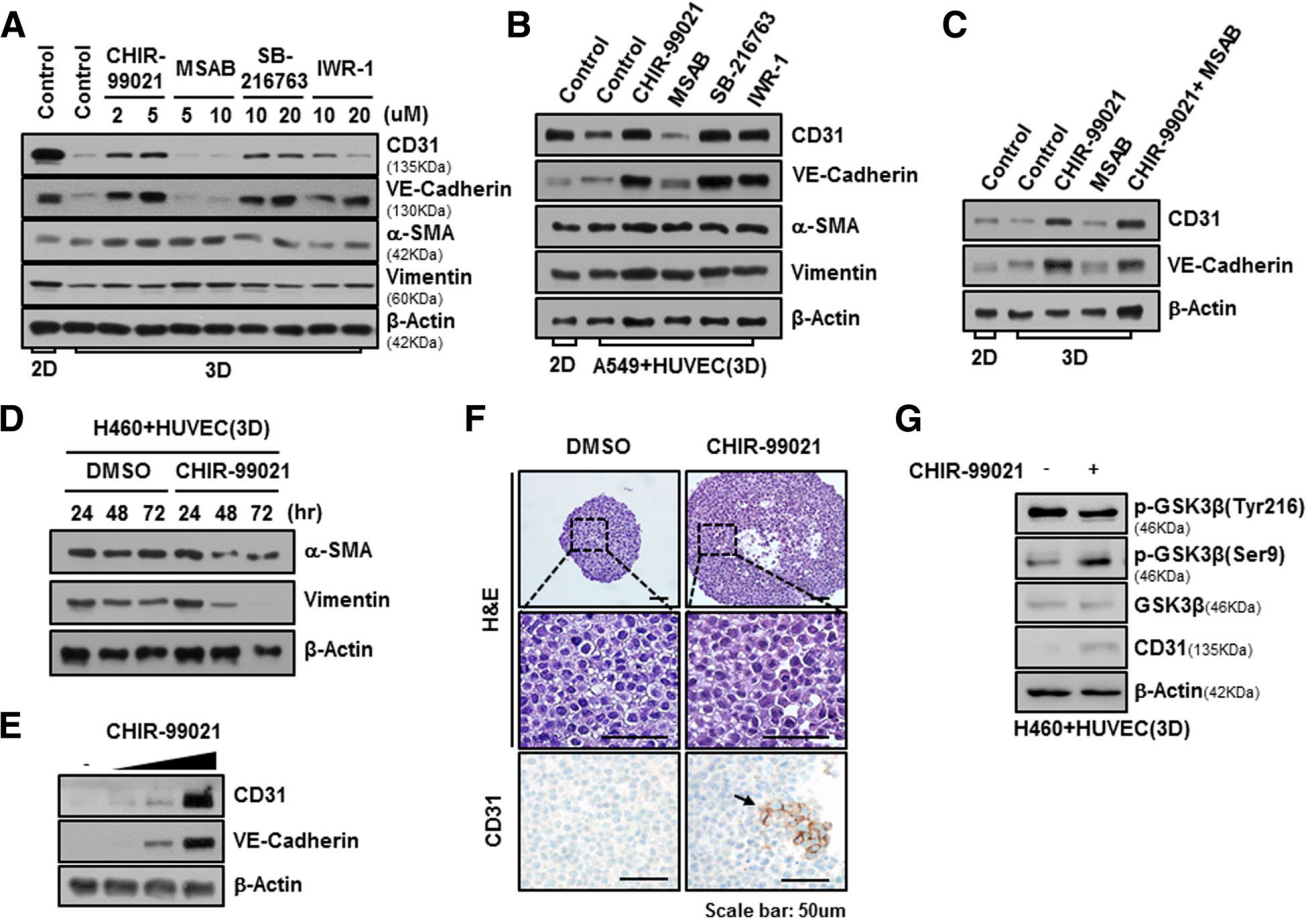
CHIR-99021, a GSK-3β inhibitor, suppresses the EndMT process. **a** NCI-H460 cells were co-cultured with HUVECs in 2D and 3D-culture systems at a ratio of 5:5, and then treated with the indicated drugs for 24 h. Cells were harvested and immunoblotted with anti-CD31, anti-VE-cadherin, anti-α-SMA, anti-vimentin, and anti-β-actin antibodies. **b** A549 were co-cultured with HUVEC under 2D and 3D condition at ratio of 5:5, and then treated with 2 μM CHIR-99021, 5 μM MSAB, 20 μM SB-216763, and 20 μM IWR-1. After 24 h, lysates were analyzed by immunoblotting using indicated antibodies. **c** NCI-H460 cells were co-cultured with HUVECs under 2D and 3D conditions and then treated with 2 μM CHIR-99021, 5 μM MSAB, and co-treated with both 2 μM CHIR-99021 and 5 μM MSAB. After 24 h, lysates were analyzed by immunoblotting using anti-CD31, anti-VE-cadherin, and anti-β-actin antibodies. **d** NCI-H460 cells were co-cultured with HUVECs under 3D conditions, and then treated with 2 μM CHIR-99021 for 24, 48, and 72 h. **e** NCI-H460 cells were co-cultured with HUVECs under 3D conditions, and then treated with 0.5, 1, and 2 μM CHIR-99021 for 24 h. Lysates were analyzed by immunoblotting. **f** Representative images of MCTSs containing NCI-H460 cells and HUVECs treated with CHIR-99021 for 1 day. Shown are spheroids stained with H&E, and immunohistochemically stained for CD31. Scale bars = 50 μm. **g** NCI-H460 cells were co-cultured with HUVECs in a 3D culture system, and then treated with 1 μM CHIR-99021 for 24 h. Lysates were analyzed by immunoblotting using anti-phospho-GSK-3β (Tyr216), anti-phospho-GSK-3β (Ser9), anti-GSK-3β, anti-CD31, and anti-β-actin antibodies

Inhibition of GSK-3β activity by CHIR-99021 and SB-216763 significantly maintained in the steady state expression of CD31 and VE-Cadherin of HUVEC cells in MCTS with NCI-H460 cells and HUVEC cells. On the other hands, inhibitors of β-catenin, MSAB and IWR-1, could not maintained expression of CD31 and VE-Cadherin of HUVEC cells in same MCTSs (Fig. 4-a). When A549 cells were used instead of NCI-H460 cells, we detected the similar effects of compounds that act against Wnt/β-catenin signaling (Fig. 4-b). We investigated effects of combination treatment CHIR-99021 and MSAB, because β-catenin is a downstream protein of GSK-3β and is regulated by GSK-3β in Wnt/β-catenin signaling pathway. Unexpectedly, inhibition of Wnt/β-catenin signaling by combination treatment of CHIR-99021 and MSAB significantly maintained in the expression of CD31 and VE-Cadherin regardless of the activity of β-catenin in MCTS with NCI-H460 cells and HUVEC cells (Fig. 4-c). Therefore, these results inferred that signal pathway of EndMT in 3D co-culture with NSCLC and HUVEC cells is not the Wnt/β-catenin signaling but the various signal pathway by GSK-3β targeted proteins.

Because EndMT leads to the upregulation of mesenchymal cell markers such as α-SMA and Vimentin, we also measured expression of α-SMA and Vimentin in MCTS grown with NCI-H460 and HUVEC cells after 24, 48 and 72 h of treatment of CHIR-99021 in order to detect exact EndMT phenomena. As a results, α-SMA and Vimentin were significantly decreased after 48 and 72 h of treatment of CHIR-99021, but not 24 h (Fig. 4-d). In addition, Treatment of CHIR-99021 during 24 h sufficiently increased expression of CD31 and VE-Cadherin in MCTS with NCI-H460 cells and HUVEC cells in a dose-dependent manner (Fig. 4-e). These results showed that CHIR-99021 induced downregulation of mesenchymal cell markers after upregulation of CD31 in MCTS with NCI-H460 and HUVEC.

To investigate the architectural change of MCTS and expression level of CD31 by treatment of CHIR-99021, we examined H&E staining and immunohistrochemistry using CD31 antibody. Interestingly, when CHIR-99021 was treated in MCTS for 24 h, spheroid size was bigger than DMSO-treated MCTS and we observed a lot of CD31 positive cells in CHIR-99021-treated MCTS (Fig. 4-f). In addition, we observed that phosphorylated GSK-3β at Tyr 216 is decreased by CHIR-99021 treatment for 24 h in 3D co-culture with NCI-H460 and HUVEC cells, whereas phosphorylated GSK-3β at Ser 9 is significantly increased in CHIR-99021-treated MCTS for 24 h (Fig. 4-g). These results indicated that CHIR-99021 inhibits EndMT process through regulation of activity of GSK-3β in MCTS with NSCLC and HUVEC.

### Co-culture with NSCLC cells and HUVEC cells to evaluate chemoresistance in MCTS

To investigate whether co-culture with NSCLC cells and HUVEC cells induces chemo-resistance in MCTS, we formed MCTS with NSCLC cells lines and stromal cells such WI38, and HUVEC.

**Fig. 5:**
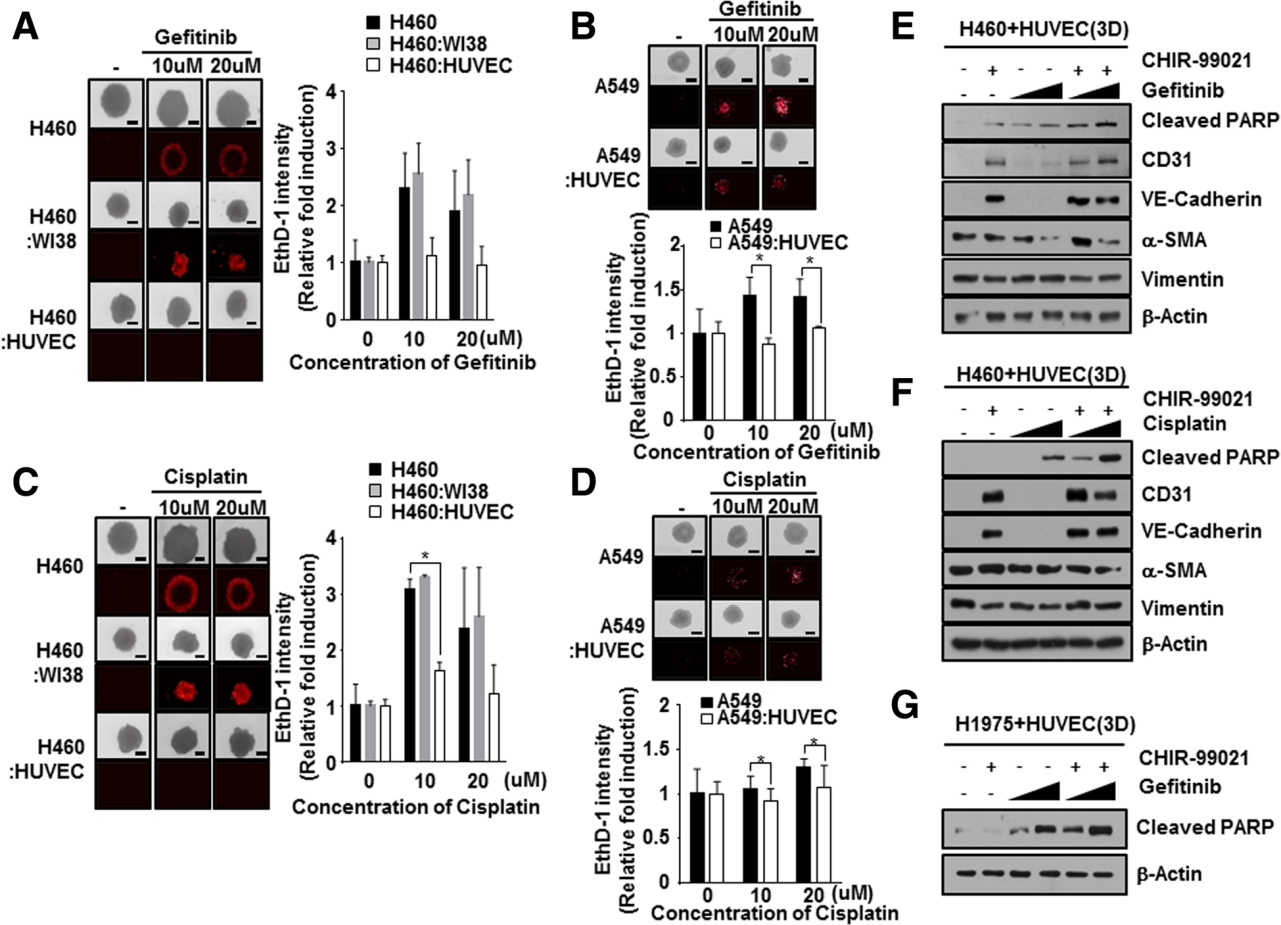
Interplay between NSCLC cells and HUVECs promotes chemoresistance in MCTSs. **a** and **b** NCI-H460 (**a**) and A549 (**b**) spheroids were co-cultured with or without stromal cells (WI38 or HUVECs) for 2 days, and then treated with 10 or 20 μM Gefitinib for 2 days. Chemoresistance was measured by staining with EthD-1, a cell death marker. The intensity of EthD-1 was analyzed and values were normalized to control (0.01% DMSO). **c** and **d** NCI-H460 (**c**) and A549 (**d**) spheroids were co-cultured with or without WI38 cells and HUVECs, and stained with EthD-1 to detect cell death, 2 days after treatment with 10 or 20 μM Cisplatin. Data are shown as mean ± SD from two independent experiments in triplicate. *p < 0.05 versus Control (NCI-H460 or A549). **e** and **f** NCI-H460 cells were co-cultured with HUVECs in a 3D culture system, and then treated with 1 μM CHIR-99021, 10 μM or 20 μM Gefitinib (**e**) or Cisplatin (**f**) alone, or a combination of both, for 24 h. Cells were harvested and immunoblotted with anti-cleaved PARP, anti-CD31, anti-VE-cadherin, anti-α-SMA, anti-vimentin, and anti-β-actin antibodies. (**g**) H1975 cells were co-cultured with HUVECs under 3D conditions, and then treated with 1 μM CHIR-99021, 10 μM or 20 μM Gefitinib alone, or a combination of both, for 24 h

NCI-H460 tumor spheroids, grown together with WI38 cells or HUVEC cells, were incubated with Gefitinib for 48 h. Cell death in MCTS, which consisted in NCI-H460 grown together with or without WI38 cells, was sufficiently increased by treatment with Gefitinib, whereas Gefitinib had less of an effect on cell death in MCTS grown together with NCI-H460 and HUVEC cells (Fig. 5-a). When A549 cells were used instead of NCI-H460 cells, MCTS grown with A549 and HUVEC cell exhibited more strong resistance to Gefitinib than A549 spheroids (Fig. 5-b). When we examined sensitivity to Cisplatin in MCTSs, MCTS grown with HUVEC cells also displayed greater resistance to Cisplatin compared to NCI-H460 spheroids and MCTS grown with NCI-H460 and WI38 cells (Fig. 5-c). MCTS grown with HUVEC cell also showed more strong resistance to Cisplatin than A549 spheroids (Fig. 5-d). These results prompted us to focus on how we could block the EndMT process and chemoresistance by crosstalk between HUVEC cells and NSCLC, because HUVEC cells could increase spheroid compactness as well as chemoresistance.

### CHIR-99021 markedly improved efficacy of chemotherapeutic drugs in NSCLC-MCTS with HUVEC

Next, we investigated whether EndMT status changes induced by pretreatment with CHIR-99021 overcame chemoresistance in NSCLC-MCTS with HUVEC. Half maximal inhibitory concentrations (IC_50_) of CHIR-99021 are 27.4 μM in NCI-H460 cells and 25.3 μM in A549 cells (Additional file 1: Figure S3).

Expression of apoptosis related marker, cleaved-PARP, was measured following treatment with EGFR TKIs, Gefitinib (first-generation EGFR TKIs), with or without CHIR-99021 in MCTS with NCI-H460 cells (EGFR WT lung cancer cell lines) and HUVEC cells. Combination treatment of CHIR-99021 significantly enhanced sensitivity to Gefitinib in MCTS with NCI-H460 cells and HUVEC cells (Fig. 5-e). We also assessed the effects of combination treatment between CHIR-99021 and Cisplatin on cell death in MCTS with NCI-H460 cells and HUVEC cells. Combination treatment of CHIR-99021and Cisplatin also dramatically increased cleaved-PARP in MCTS with NCI-H460 cells and HUVEC cells (Fig. 5-f). H1975 cells that intrinsically harbor EGFR L858R/T790 M were used to generate lung MCTS with HUVEC instead of NCI-H460 cells. Surprisingly, combination treatment of low dose of Gefitinib and CHIR-99021 elevated expression of cleaved-PARP versus single treatment of Gefitinib in MCTS grown with H1975 cells and HUVEC cells (Fig. 5-g). These results suggested that CHIR-99021 can overcome the resistance to EGFR TKIs in 3D tumor microenvironments of NSCLC regardless of cell types of EGFR mutation. These results indicated that CHIR-99021 markedly increases chemo-sensitivity in NSCLC-MCTS with HUVEC cells.

### CHIR-99021 enhances the efficiency of anticancer therapies in vivo

**Fig. 6:**
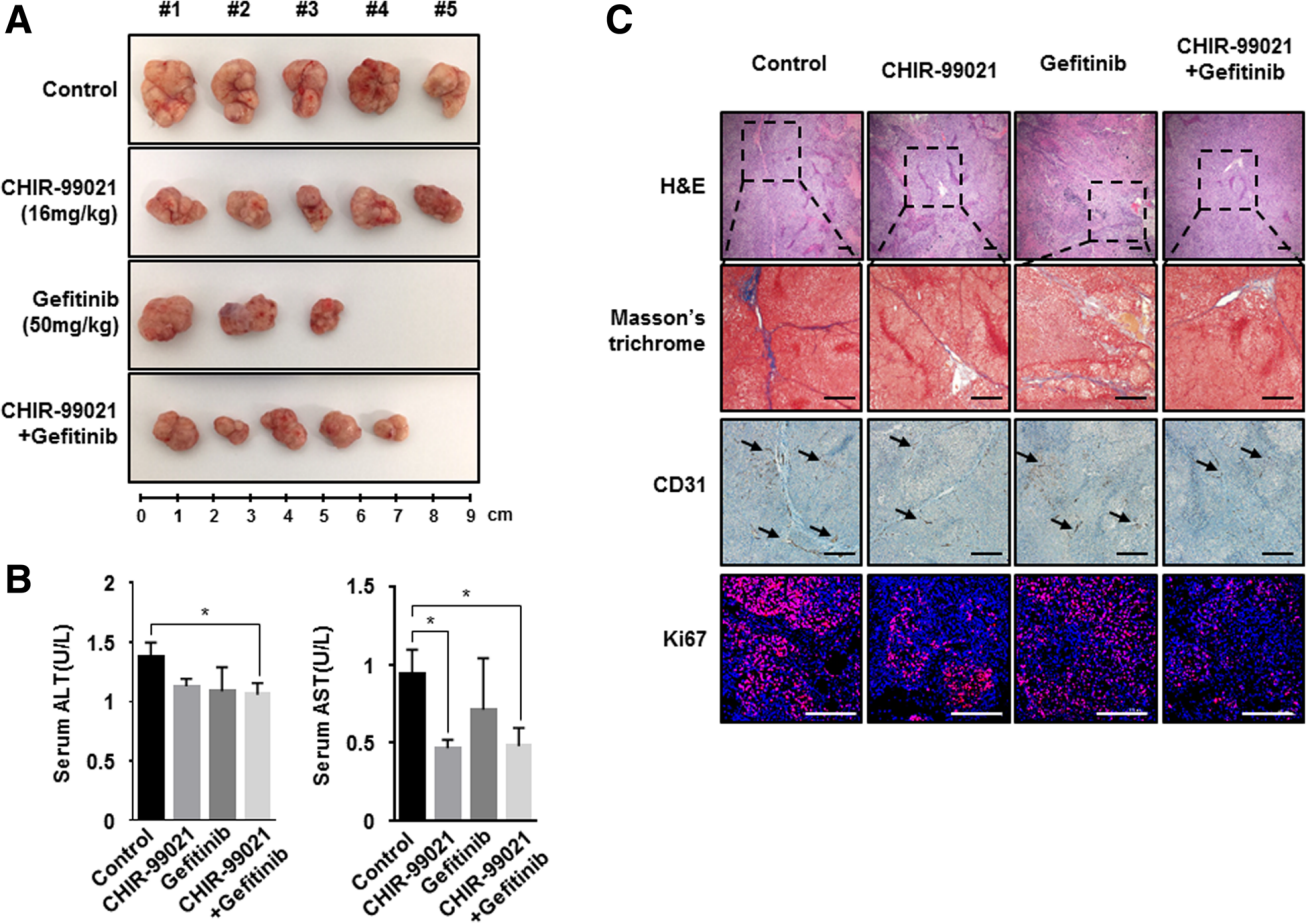
CHIR-99021, a GSK-3β inhibitor, suppresses the EndMT process and overcomes chemoresistance in MCTSs. **a** Representative images of tumor size after 12 days of treatment as indicated. Tumor volumes of MCTS in BALB/c-nu mice after treatment with 16 mg/kg CHIR-99021, 50 mg/kg Gefitinib, or combination of CHIR-99021 and Gefitinib. Drug administration was initiated once the tumor reached a size of 100–200 mm^3^. **b** Serum ALT and AST levels, measured in MCTS xenograft mice after 12 days post-treatment. **c** Representative images of H&E-stained tumors, as well as immunohistochemical analysis using Masson’s trichrome staining and antibodies against CD31 and Ki67, in the tumors of MCTS xenograft mice after treatment with the indicated drugs. The black arrow indicates the CD31-positive area. Results are presented as mean ± standard error of the means. *P < 0.05, **P < 0.01, *** *P* < 0.001 versus Control. Scale bars = 200 μm

To determine whether CHIR-99021 could sensitize enhances the efficiency of anticancer therapies in vivo system, we generated xenograft mouse model that injected MCTS with NCI-H460 cells and HUVEC cells to BALB/c-nu mice. Administration of CHIR-99021 showed just a subtle reduction of tumor growth and Gefitinib alone also induced tumor regression. However, treatment of administration of Gefitinib induced the sudden death of some mice. The other side, Gefitinib plus treatment with CHIR-99021 significantly inhibited tumor volume, versus mice treated with Gefitinib alone without sudden death of mice (Fig. 6-a). These data suggested that CHIR-99021 could be sensitizer for highly efficient treatment of Gefitinib.

Recently, there are several reports that hepatotoxicity were observed during long-term Gefitinib administration in patients with non-small-cell lung cancer [32–35]. Because we observed sudden death of mice during the administration of Gefitinib, we evaluated serum alanine aminotransferase (ALT) activity and aspartate aminotransferase (AST) values for hepatotoxicity assessment of experiment object mice in Fig. 6-a. No significant differences were observed in the levels of AST and ALT between CHIR-99021 treated mice, Gefitinib treated mice, Gefitinib + CHIR-99021 treated mice and saline treated mice (Fig. 6-b). This result indicated that the treatment of Gefitinib and Gefitinib + CHIR-99021 was not hepatotoxic in xenograft mice.

Next, we observed alteration of fibrotic degree by treatment of CHIR-99021. Masson’s trichrome staining showed that treatment of CHIR-99021, Gefitinib + CHIR-99021 more significantly attenuated fibrotic degree than treatment of Gefitinib in MCTS with NCI-H460 cells and HUVEC cells implanted xenograft mice (Fig. 6-c, upper). Antibody to CD31 was primarily used to confirm the presence of vascular endothelial cells in histological tissue sections. Unexpectedly, CD31 was distributed irregularly over a relatively large area in tumor tissues from none or Gefitinib treated mice, whereas tumor tissues from CHIR-99021 or combination treated mice showed that expression of CD31 was mildly decreased (Fig. 6-c, middle). In addition, we also performed immunofluorescence staining using anti-Ki67 in tumor tissues to measure tumor cell proliferation around blood vessels. As a result, Ki67-positive cells decreased in tumor tissues from CHIR-99021 or combination treated mice compared with tumor tissues from none or Gefitinib treated mice (Fig. 6-c, lower).

Taken together, the results showed that treatment with CHIR-99021 inhibits EndMT, thereby facilitating the robust therapeutic activity of combined CHIR-99021 and anticancer therapies in human lung cancer.

## Discussion

Lung cancer continues to be the most common cause of cancer-related mortality worldwide. Between 52 and 58% of lung cancer patients present with advanced-stage disease, and a vast majority of these patients do not survive despite treatment. Similarly, the prognosis remains poor even in locally advanced disease because of the high relapse rate and early formation of micrometastases [36]. Recent advances in molecular diagnostics and targeted agents, such as immunotherapeutics, have propelled the rapid development of novel treatment modalities for lung cancer. However, treatment with targeted agents inevitably leads to drug resistance [37]. Hence, deep knowledge of resistance mechanisms and the development of novel agents or strategies targeting resistant tumors are sorely needed. In a previous study, crosstalk between cancer cells and stromal cells, such as hepatic stellate cells, fibroblasts, vascular endothelial cells, and ECM-related proteins, was found to promote chemoresistance and migration of hepatocellular carcinoma in MCTSs [38]. Hence, we believe that control of tumor microenvironment-mediated drug resistance might represent a promising therapeutic strategy for cancer therapy.

Generally, endothelial cells that have changed morphologically through the endothelial-to-mesenchymal transition (EndMT) or CAFs may play pivotal roles in inflammatory diseases and fibrosis in various tissues. Based on recent studies, we hypothesized that EndMT may occur in tumor microenvironments naturally without stimuli such as chemical or radiation etc. In recent years, a paradigm shift from 2D to 3D cell culture techniques has occurred, because 2D cell culture involves growing cells in a flat dish, which can lead to the formation of unnatural cell attachments. Further, simplifying the 2D assay system does not provide us the data that would be utilized in translational research. Hence, we conducted comparative studies of the effects of secretomes on the activation of EndMT in HUVECs in 2D and 3D cultures of NSCLC cells. Here, we observed significant activation of the EndMT process in HUVECs in 3D co-culture, compared to that in 2D co-culture, with NSCLC cells, because 3D cell culture could better mimic the growth characteristics and microenvironment of solid tumors in vivo than a monolayer culture (Fig. 1; Additional file 1: Figure S1). These results indicate that the increase in certain growth factors in NSCLC-3D culture conditions may lead to the activation of the EndMT process in HUVECs.

We also ascertained that the paracrine effects of NSCLC cells on the activation of the EndMT process in HUVECs should be investigated in 3D culture conditions. The most common growth factors that control EndMT activation belong to the TGF-β super family of proteins, which includes isoforms TGF-β1 and TGF-β2, as well as bone morphogenetic proteins (BMPs) BMP2, BMP4, BMP6, BMP9, and BMP10 [39]. Other growth factors have also been reported recently, such as FGFs [40], HGF [41] and CTGF [42]. However, there was no difference in the secretion of TGF-β between 2D- or 3D-cultured NSCLC cells (Fig. 3-A, B). These results indicate that signals other than TGF-β trigger the EndMT process in HUVECs in 3D culture conditions.

Here, we created MCTSs containing NSCLC cells and stromal cells, which are fibroblasts and endothelial cells, in order to mimic tumor complexity in vitro (Fig. 2), and observed that the ruggedness of the MCTSs was variable depending on the type of stromal cells. We were particularly intrigued by the realistic reciprocal crosstalk between NSCLC cells and HUVECs in MCTSs – co-cultures of NSCLC cells and HUVECs revealed that both cell types exhibited enhanced resistance to anticancer drugs such as Gefitinib and Cisplatin and the compactness of spheroids in MCTSs (Fig. 5). The most common cytokines to active EndMT belong to the TGF-β super family of proteins, but other signaling pathways have also been recently reported, such as Wnt/β-catenin [43], Notch [44], HSPB1 [26] and various receptor tyrosine kinases [45]. Wnt also cooperates with BMP and TGF-β through the co-occupancy of SMAD target enhancers by Wnt-activated LEF1 (lymphoid enhancer-binding factor 1; also known as TCF1α) [46, 47]. Our data indicate that the Wnt signal cascade, particularly the activity of GSK-3β, contributes to the activation of the EndMT of HUVECs in MCTSs (Figs. 3, 4). Indeed, inactivation of GSK-3β has been observed in various tumors [48–50]. Several published studies report that EndMT signaling shares some of the same pathways and effectors with the epithelial–mesenchymal transition (EMT) [51]. GSK-3β is known to regulate tumor migration and invasion through control of EMT. Signaling pathways that inactivate GSK-3β, such as phosphatidylinositol 3 kinase/Akt and mitogen-activated protein kinase, may promote the cell cycle, anti-apoptosis, and invasion, thus facilitating tumor progression [52–55]. However, our data is not discern which cell type is expressing/activates GSK-3β in MCTSs. Hence, further experiments to demonstrate specific activation of GSK-3β in HUVEC induced by co-culture with lung cancer cells in MCTSs should be performed. Likewise, all the molecular and phenotypic (apoptosis) effects of the GSK-3β inhibitor should be demonstrated to occur in HUVEC, and not in the co-cultured lung cancer cells. In terms of anticancer effects, CHIR-99021 is very effective to control tumor growth, as is Gefitinib. And inactivation of GSK-3β by combined treatment with CHIR-99021 reinforces sensitivity to Cisplatin and Gefitinib in MCTSs in vitro as well as in MCTS xenograft mice, through inhibition of EndMT (Fig. 5-e, f, g; Fig. 6).

## Conclusions

Our results provide clear evidence that reciprocal crosstalk between NSCLC cells and HUVECs leads to the endothelial-to-mesenchymal transition via the activation of GSK-3β in multicellular tumor spheroid models, and thereby plays a central role in resistance to lung cancer therapy. Therefore, our results suggest that combined treatment with GSK-3β inhibitor and conventional chemotherapy may be a promising approach to overcome environment-mediated drug resistance and for the treatment of EndMT-related disorders in lung cancer.

## Additional files

Additional file 1: **Figure S1.** NCI-H460 cells were cultured under 2D and 3D condition using the same number of cells with the same amount of media. After 3 days, the conditional media (CM) was mixed with HUVEC original media at indicated ratio and then treated HUVEC cells for 24 and 48 h. Representative image of immunofluorescence staining for CD31 (green), α-SMA (red), and nuclei (blue) in HUVEC cells. **Figure S2.** (A) Target names of spots in Human Growth Factor Antibody Array. (B) Human Growth Factor Antibody Array were used to measure the level of growth factor in samples from 2D and 3D co-cultured NCI-H460 with HUVEC. **Figure S3.** Dose response curve for cell viability in NCI-H460 or A549 cells following treatment CHIR-99021 for 48 h. (PDF 499 kb)

Additional file 2: The raw data from microarray. (XLSX 389 kb)

## Abbreviations

2D: Two dimensional
3D: Three dimensional
ALT: Alanine aminotransferase
AST: Aspartate aminotransferase
CAFs: Cancer-associated fibroblasts
CM: Conditioned medium
CTGF: Connective tissue growth factor
ECM: Extracellular matrix
EGFR: Epidermal growth factor receptor
EndMT: Endothelial-to-mesenchymal transition
EthD-1: Ethdium homodimer-1
GSK-3β: Glycogen synthase kinase -3β
HUVEC: Human Umbilical Vein Endothelial Cells
MCTSs: Multicellular tumor spheroids
NSCLC: Non-small-cell lung cancer cells
PI3K: Phosphatidylinositol-4,5-bisphosphate 3-kinase
PTEN: Phosphatase and tensin homolog
TGF-β: Transforming growth factor-β
TME: Tumor microenvironments
α-SMA: α-smooth muscle actin
