# Supplementary material for: GSK-3β regulates the endothelial-to-mesenchymal transition via reciprocal crosstalk between NSCLC cells and HUVECs in multicellular tumor spheroid models
Source: J Exp Clin Cancer Res. 2019 Feb 1;38:46. doi: 10.1186/s13046-019-1050-1 (PMC6359813; doi:10.1186/s13046-019-1050-1)

Supplementary Figure legends

Supplementary Figure 1.

NCI-H460 cells were cultured under 2D and 3D condition using the same number of cells with the same amount of media. After 3 days, the conditional media (CM) was mixed with HUVEC original media at indicated ratio and then treated HUVEC cells for 24 and 48 hr. Representative image of immunofluorescence staining for CD31 (green), α-SMA (red), and nuclei (blue) in HUVEC cells.

Supplementary Figure 2.

(A) Target names of spots in Human Growth Factor Antibody Array. (B) Human Growth Factor Antibody Array were used to measure the level of growth factor in samples from 2D and 3D co-cultured NCI-H460 with HUVEC.

Supplementary Figure 3

Dose response curve for cell viability in NCI-H460 or A549 cells following treatment CHIR-99021 for 48 hr.

Supplementary Figure 1

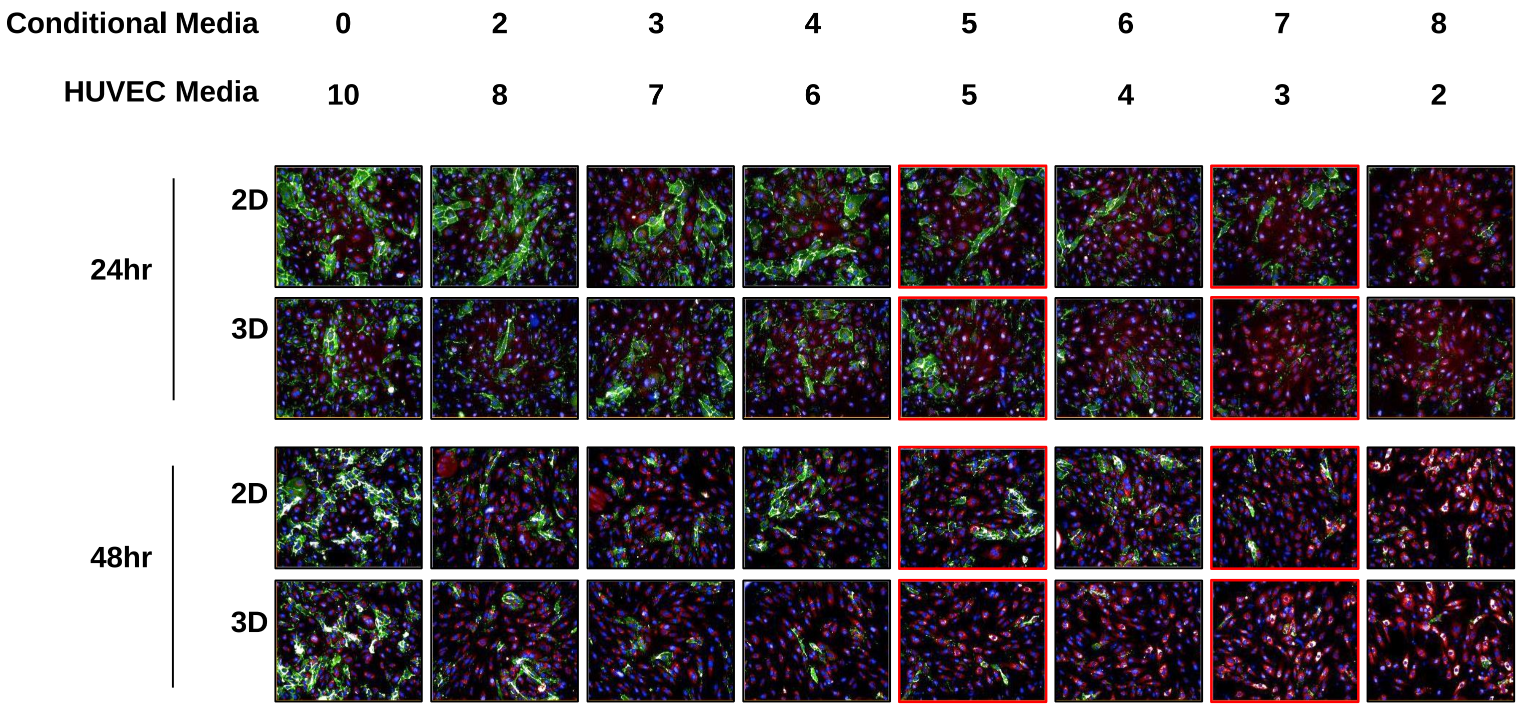

Supplementary Figure 2

A.

Human Growth Factor Antibody Array

Each antibody is spotted in duplicate vertically

	A	B	C	D	E	F	G	H	I	J	K	L
1	POS	POS	NEG	NEG	AR	b-FGF	β-NGF	EGF	EGFR	FGF-4	FGF-6	FGF-7
2												(KGF)
3	GCSF	GDNF	GM-CSF	HB-EGF	HGF	IGFBP-1	IGFBP-2	IGFBP-3	IGFBP-4	IGFBP-6	IGF-1	IGF-1R
4												
5	IGF-2	M-CSF	M-CSFR	NT-3	NT-4	PDGFR-α	PDGFR-β	PDGF-AA	PDGF-AB	PDGF-BB	PLGF	SCF
6												
7	SCFR	TGF-α	TGF-β1	TGF-β2	TGF-β3	VEGF-A	VEGFR2	VEGFR3	VEGF-D	BLANK	BLANK	POS
8	(CD117)											

B.

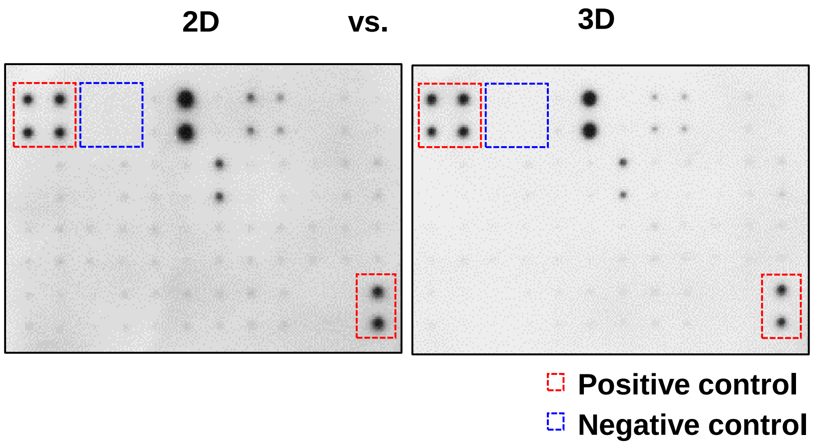

C.

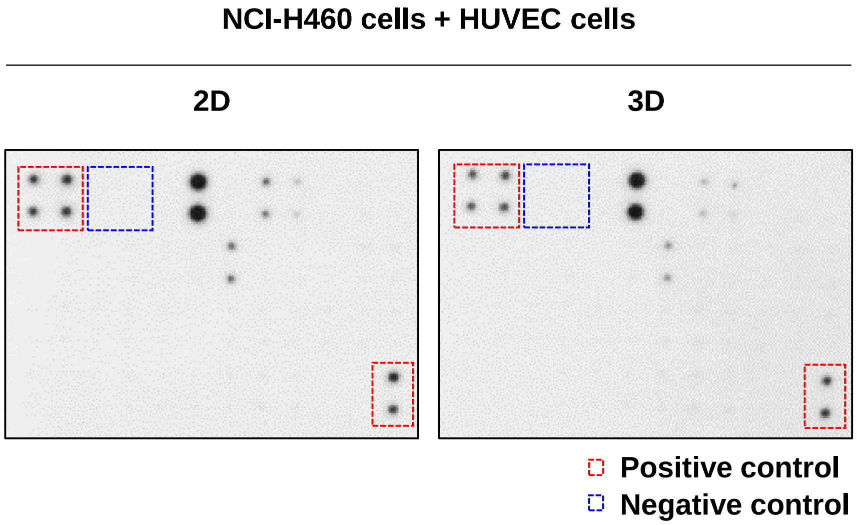

Supplementary Figure 3

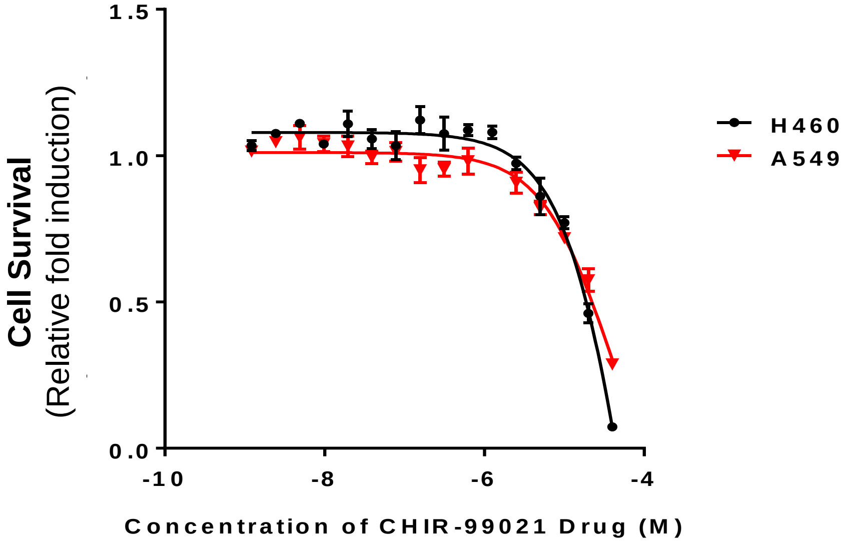

Supplement: Supplementary file 1 — Figure S1. NCI-H460 cells were cultured under 2D and 3D condition using the same number of cells with the same amount of media. After 3 days, the conditional media (CM) was mixed with HUVEC original media at indicated ratio and then treated HUVEC cells for 24 and 48 h. Representative image of immunofluorescence staining for CD31 (green), α-SMA (red), and nuclei (blue) in HUVEC cells. Figure S2. (A) Target names of spots in Human Growth Factor Antibody Array. (B) Human Growth Factor Antibody Array were used to measure the level of growth factor in samples from 2D and 3D co-cultured NCI-H460 with HUVEC. Figure S3. Dose response curve for cell viability in NCI-H460 or A549 cells following treatment CHIR-99021 for 48 h. (PDF 499 kb) [file 13046_2019_1050_MOESM1_ESM.pdf]
